# Thiazides and Risk of Hyponatremia by Age and Sex

**DOI:** 10.1001/jamanetworkopen.2026.4642

**Published:** 2026-04-02

**Authors:** Cecilia Bergh Fahlén, Henrik Falhammar, Jakob Skov, Jonatan D. Lindh, Buster Mannheimer

**Affiliations:** 1 Department of Clinical Science and Education at Södersjukhuset, Karolinska Institutet, Stockholm, Sweden; 2 Department of Internal Medicine, Section of Diabetes and Endocrinology, Södersjukhuset, Stockholm, Sweden; 3 Department of Molecular Medicine and Surgery, Karolinska Institutet, Karolinska University Hospital, Stockholm, Sweden; 4 Department of Endocrinology, Karolinska University Hospital, Stockholm, Sweden; 5 Department of Medicine, Karlstad Central Hospital, Karlstad, Sweden; 6 Department of Laboratory Medicine, Division of Clinical Pharmacology, Karolinska Institutet, Stockholm, Sweden; 7 Department of Clinical Pharmacology, Karolinska University Hospital, Stockholm, Sweden

## Abstract

**Importance:**

Thiazide diuretics are a cornerstone for the treatment of hypertension, but their use is associated with development of hyponatremia. Women and older adults are particularly vulnerable, but population-based estimates of absolute risk are largely absent. Such data are a prerequisite for a robust risk-benefit assessment in the clinical setting.

**Objective:**

To compare new use of thiazide diuretics with calcium channel blockers (CCBs) and subsequent risk of hyponatremia among different age groups and between the sexes.

**Design, Setting, and Participants:**

This propensity score–matched cohort study included 159 080 individuals 18 years or older in the Stockholm Sodium Cohort, a research database established to investigate the association between thiazide and hyponatremia among individuals in Stockholm, Sweden, between July 1, 2006, and December 31, 2018. Statistical analysis was performed between January 2025 and January 2026.

**Exposure:**

Newly initiated treatment with thiazide diuretics and CCBs.

**Main Outcomes and Measures:**

The primary outcome was profound hyponatremia (ie, a sodium concentration <125 mEq/L). Secondary outcomes were sodium concentrations less than 130 mEq/L and less than 135 mEq/L.

**Results:**

A total of 79 540 individuals (median age, 63 years [IQR, 54-72 years]; 41 275 women [51.9%]) initiating thiazide treatment were propensity score matched with 79 540 individuals (median age, 63 years [IQR, 54-72 years]; 41 168 women [51.8%]) receiving CCBs. The cumulative incidence of profound hyponatremia was 0.80% (95% CI, 0.74%-0.87%) for thiazide users and 0.46% (95% CI, 0.41%-0.51%) for CCB users during the first 2 years of treatment. The occurrence of profound hyponatremia with thiazide treatment was higher among women (cumulative incidence, 1.04% [95% CI, 0.94%-1.15%) and individuals 80 years or older (cumulative incidence, 2.40% [95% CI, 2.07%-2.73%]). Thus, among women 80 years or older, the number needed to harm (NNH) was 53 (95% CI, 41-73) for developing sodium concentrations less than 125 mEq/L, 28 (95% CI, 22-38) for concentrations less than 130 mEq/L, and 16 (95% CI, 13-20) for concentrations less than 135 mEq/L. This was in marked contrast with women younger than 65 years, for whom the corresponding NNH was 790 (95% CI, 408-11 966) for developing sodium concentrations less than 125 mEq/L, 818 (95% CI, 303-∞) for concentrations less than 130 mEq/L, and 120 (95% CI, 78-261) for concentrations less than 135 mEq/L.

**Conclusions and Relevance:**

In this cohort study comprising 159 080 individuals, the association between newly initiated thiazide diuretics and hyponatremia was negligible among individuals younger than 65 years of age. In contrast, among older adults, especially among women, the association was substantial. The results may incentivize the prescriber toward choosing an alternative antihypertensive treatment. If thiazides are initiated, subsequent monitoring of serum sodium concentrations should be considered.

## Key Points

**Question:**

What is the age-specific and sex-specific risk of hyponatremia associated with newly initiated thiazides?

**Findings:**

In this cohort study of 159 080 individuals, among women 80 years or older, use of thiazide diuretics was associated with a statistically and clinically significant risk of hyponatremia among women aged 80 years or older compared with use of calcium channel blockers. In contrast, among women younger than 65 years, the risk of hyponatremia was negligible.

**Meaning:**

The findings of this study suggest that prescribers should consider close clinical monitoring among older adults, especially women, receiving thiazide, and that alternative antihypertension treatments may be considered in this population.

## Teaser

This cohort study compares risk of hyponatremia among patients receiving thiazide diuretics vs calcium channel blockers by age and sex.

## Introduction

Thiazide diuretics are a cornerstone for the treatment of hypertension, a strong risk factor for the development of cardiovascular disease.^1,2^ Thiazide diuretics are effective, low cost, and often well tolerated and therefore a good choice for first-line treatment. However, they may also cause adverse effects, such as hyponatremia.^3,4,5^ Hyponatremia is often caused by medication^6^ and associated with a large variety of symptoms, ranging from fatigue and confusion to headache, nausea, and sometimes seizures.^7^ The mechanisms underlying thiazide-induced hyponatremia are poorly understood but are thought to involve a combination of excessive fluid intake, osmotic inactivation of sodium, and decreased ability to excrete excess free water.^4^ Data indicate that 3 in 10 patients taking thiazide diuretics develop hyponatremia at some point.^8^ Observational studies assessing relative risks indicate that the risk disproportionally affects older individuals and females.^3,4,9^ With rare exceptions,^10^ population-based studies investigating absolute risks are, however, lacking. Such data are a prerequisite for a robust risk-benefit assessment in the clinical setting.

In this study we used data from the Stockholm Sodium Cohort (SSC), a research database to investigate the association between thiazide treatment and hyponatremia.^11^ We compared new use of thiazide diuretics with the initiation of an alternative antihypertensive drug, calcium channel blockers (CCBs), and subsequent risk of hyponatremia.

## Methods

This was a cohort study comparing the occurrence of hyponatremia among residents of Stockholm, Sweden, aged 18 years or older. The design was intention to treat. Individuals initiating treatment with thiazide diuretics (Anatomical Therapeutic Chemical [ATC] codes C03AA, C09BA, C09DA, and C03EA01) or CCBs (ATC codes C08C, C09BB, and C09DB) between July 1, 2006, and December 31, 2018, were included. Individuals dispensed a combination of CCBs and thiazides (ATC code C08GA) and those with an index date before July 1, 2006, were excluded. In addition, individuals with end of data within 2 years after the index date were excluded. A flowchart of the study population is presented in the eFigure in Supplement 1. The study was approved by the Swedish Ethical Review Authority; informed consent was waived because this was a retrospective study, and it was not feasible to obtain consent from such a large number of individuals. The study followed the Strengthening the Reporting of Observational Studies in Epidemiology (STROBE) reporting guideline for cohort studies.

The study was based on the SSC, a research register that includes information on all inhabitants of the Stockholm region who at any time between the years 2005 and 2018 had a serum sodium concentration analyzed at any of the 3 laboratories in Stockholm responsible for biochemical tests performed during routine health care controls. For included individuals, a wide range of data was extracted for the entire time period. The SSC includes all test results across 100 biochemical parameters for all patients (approximately 1 600 000). The register also holds data from major Swedish health and population registers with socioeconomic data, health care contacts, pharmaceutical drug use, and causes of death, as well as inpatient and specialist outpatient diagnoses. The coverage is modest among younger and healthier individuals but reaches 92% among patients older than 65 years and 97% among patients older than 80 years.^11^

All individuals in the SSC were eligible for inclusion. If the first thiazide dispensation was preceded by a minimum period of 1 year without thiazide dispensation, the exposure was considered new. If there were several new thiazide prescriptions, only the first prescription was included in the study. The index date was defined as the date of the first dispensation of thiazide medication or CCB.

Sodium concentrations were adjusted for plasma glucose concentrations when checked within 24 hours of the serum sodium, using the equation by Hillier et al.^12^ If no glucose concentrations were available, unadjusted sodium concentrations were used. The primary outcome was defined as an event of profound hyponatremia (sodium concentration <125 mEq/L [to convert to millimoles per liter, multiply by 1.0]) occurring after the index date. In addition, sodium concentrations less than 130 mEq/L and less than 135 mEq/L were included as secondary outcomes.

Data on relevant comorbidities by index date, including cardiovascular, infectious, metabolic, and gastrointestinal diseases, were collected from the SSC and originally derived from the National Patient Register, with diagnoses coded according to the *International Statistical Classification of Diseases and Related Health Problems, Tenth Revision* (*ICD-10*). The National Patient Register collects data from discharge diagnoses from hospital wards and specialist outpatient clinics.^13^ For chronic diseases, any baseline diagnosis (since 1997, when *ICD-10* codes began to be used in Sweden, until the index date) was considered. For ischemic heart disease and cerebrovascular disease, *ICD-10* codes registered less than 90 days from the index date were separated from codes registered 90 days or more from the index date. The variables of infection and acute pulmonary embolism included *ICD-10* codes only registered less than 90 days from the index date. For the remaining variables, *ICD-10* codes registered at any time were included. Information on use of medications derived from the Swedish Prescribed Drug Register associated with hyponatremia coded according to the ATC classification was also included. Drug exposure was defined as drug dispensation less than 120 days prior to the index date. Data on education and income were collected from the longitudinal integrated database for health insurance and labor market studies.^14^

### Statistical Analysis

Statistical analysis was performed from January 2025 to January 2026. For the analysis of the association between thiazide exposure and hyponatremia, the cumulative incidence of hyponatremia was compared between individuals initiating treatment with a thiazide or CCB. To achieve balanced groups with regard to potential confounders, generalized full 1:1 propensity score matching was performed based on age, sex, and a wide array of comorbidities and medications (defined in eTable 1 in Supplement 1), along with standardized mean differences. Propensity scores were estimated using logistic regression, and individuals were matched 1:1 using a fine-grained propensity score subclassification (77 806 subclasses), with random selection of 1 control per thiazide user within each subclass.

Descriptive statistics included percentages and median (IQR) values as appropriate. Subgroup analyses were performed based on sex and age (<65 years, 65-79 years, and ≥80 years). Cumulative incidences of hyponatremia (<125 mEq/L in the primary analysis; <130 mEq/L and <135 mEq/L in secondary analyses) were calculated separately for the 2 study groups by means of the Kaplan-Meier estimator, with death, end of follow-up, and end of data treated as the only censoring events. Based on these estimators, relative risks (RRs) and absolute risk differences were calculated separately at 4 different time points (14, 30, 90, and 730 days). The number of individuals treated per case of hyponatremia was calculated as the inverse of the absolute risks. The number needed to harm (NNH) was calculated as the inverse of the absolute risk difference between individuals treated with CCBs and those treated with thiazides. The potential influence of death as a competing risk was investigated by means of cause-specific hazard models with death (regardless of cause) as the outcome and hyponatremia as a censoring event. To detect residual bias, the main analysis was repeated using death (regardless of cause, frequency not expected to be increased by thiazides) as the outcome, without censoring at hyponatremia events. In addition to the intention-to-treat analysis, a per-protocol analysis, excluding study participants with a period of more than 6 months between subsequent drug dispensations was also performed in subgroups based on sex and age (<65 years, 65-79 years, and ≥80 years).

All analyses were performed in R, version 4.3.1 (packages data.table, MatchIt, quickmatch, survival, and survminer; R Project for Statistical Computing). The threshold for statistical significance was 2 sided, and *P* < .05 was considered statistically significant.

## Results

In this cohort study, 116 734 individuals (median age, 63 years [IQR, 54-72 years]; 63 799 women [54.7%] and 52 935 men [45.3%]) initiated bendroflumethiazide (BFT) or hydrochlorothiazide (HCT) treatment, and 184 841 (median age, 63 years [IQR, 53-72 years]; 89 187 women [48.3%] and 95 654 men [51.7%]) initiated CCB treatment (Table 1). Propensity score matching (1:1) was successful for 79 540 thiazide-exposed patients (median age, 63 years [IQR, 54-72 years]; 41 275 women [51.9%] and 38 265 men [48.1%]) and thus resulted in the inclusion of 79 540 CCB-exposed patients (median age, 63 years [IQR, 54-72 years]; 41 168 women [51.8%] and 38 372 men [48.2%]). Other covariates were also well balanced after matching. After matching, among the individuals receiving thiazides, 14 425 received BFT and 65 115 received HCT. Baseline characteristics at the index date before and after propensity score matching are presented in Table 1.

**Table 1.** Baseline Characteristics of Adults With Newly Initiated Thiazide and CCB Treatment, Before and After Propensity Score Matching

Characteristic	Unmatched		After matching		Standardized mean difference^a^	
	Thiazides (n = 116 734)	CCBs (n = 184 841)	Thiazides (n = 79 540)	CCBs (n = 79 540)	Before matching	After matching
Age, median (IQR), y	63 (54 to 72)	63 (53 to 72)	63 (54 to 72)	63 (54 to 72)	−0.061	−0.002
Sex, No. (%)						
Women	63 799 (55)	89 187 (48)	41 275 (52)	41 168 (52)	0.128	0.003
Men	52 935 (45)	95 654 (52)	38 265 (48)	38 372 (48)	0.128	0.003
Comorbidities, No. (%)						
Malignant neoplasm	15 448 (13)	31 481 (17)	11 561 (15)	11 567 (15)	0.106	0.000
Diabetes	12 905 (11)	21 582 (12)	9293 (12)	9273 (12)	0.020	−0.001
IHD ≥90 d	7935 (7)	16 406 (9)	6120 (8)	6259 (8)	0.077	0.007
Atrial fibrillation	6438 (6)	11 601 (6)	4684 (6)	4675 (6)	0.032	0.000
CVD ≥90 d	5904 (5)	10 826 (6)	4262 (5)	4240 (5)	0.035	−0.001
Chronic pulmonary disease other than COPD	5367 (5)	10 461 (6)	4003 (5)	3983 (5)	0.048	−0.001
COPD	5450 (5)	9763 (5)	3934 (5)	3873 (5)	0.028	−0.004
Alcohol or drug abuse	4363 (4)	10 598 (6)	3500 (4)	3419 (4)	0.094	−0.005
Congestive heart failure	3472 (3)	7428 (4)	2661 (3)	2761 (4)	0.057	0.007
Hypothyroidism	3411 (3)	6836 (4)	2538 (3)	2457 (3)	0.043	−0.006
CVD <90 d	1511 (1)	4745 (3)	1249 (2)	1254 (2)	0.093	0.001
Rheumatic disease	1573 (1)	3713 (2)	1230 (2)	1177 (2)	0.052	−0.005
Liver disease	1465 (1)	3456 (2)	1129 (1)	1146 (1)	0.050	0.002
Renal disease	2420 (2)	10 010 (5)	2147 (3)	2279 (3)	0.177	0.010
Smoking	947 (0.8)	3050 (2)	825 (1)	822 (1)	0.076	0.000
Pancreatic disease	1025 (0.9)	2150 (1)	756 (1)	768 (1)	0.028	0.002
Dementia	933 (0.8)	2047 (1)	683 (0.9)	679 (0.9)	0.032	−0.001
IHD <90 d	522 (0.4)	2991 (2)	495 (0.6)	549 (0.7)	0.116	0.008
Inflammatory bowel disease	564 (0.5)	1297 (0.7)	447 (0.6)	435 (0.5)	0.029	−0.002
Pneumonia <90 d	162 (0.1)	690 (0.4)	138 (0.2)	154 (0.2)	0.046	0.005
Gastroenteritis	113 (0.1)	466 (0.3)	90 (0.1)	97 (0.1)	0.037	0.003
Adrenal failure	96 (0.1)	250 (0.1)	68 (0.1)	62 (0.1)	0.016	−0.003
Acute pulmonary embolism <90 d	71 (0.1)	342 (0.2)	67 (0.1)	72 (0.1)	0.035	0.002
Sepsis <90 d	52 (0.0)	330 (0.2)	46 (0.1)	43 (0.1)	0.040	−0.002
Cerebral infection <90 d	25 (0.0)	85 (0.0)	16 (0.0)	20 (0.0)	0.106	0.003
Malnutrition	21 (0.0)	75 (0.0)	18 (0.0)	18 (0.0)	0.013	0.000
Medications, No. (%)						
RAS inhibitors	59 891 (51)	75 443 (41)	39 561 (50)	39 831 (50)	−0.212	0.007
β-Blockers	27 209 (23)	44 297 (24)	19 019 (24)	19 075 (24)	0.015	0.002
Lipid-lowering agents	21 932 (19)	34 414 (19)	15 276 (19)	15 213 (19)	−0.004	−0.002
Proton pump inhibitors	13 119 (11)	23 385 (13)	9432 (12)	9333 (12)	0.044	−0.004
Antidepressants	11 123 (10)	18 711 (10)	7834 (10)	7644 (10)	0.020	−0.008
Opioids	9902 (9)	16 610 (9)	6973 (9)	6905 (9)	0.018	−0.003
Furosemide	4435 (4)	8791 (5)	3463 (4)	3480 (4)	0.047	0.001
Antiepileptics	2477 (2)	4853 (3)	1870 (2)	1835 (2)	0.033	−0.003
Quinolones	1793 (2)	2919 (2)	1205 (2)	1196 (2)	0.003	−0.001
Antipsychotics	1519 (1)	2922 (2)	1083 (1)	1081 (1)	0.023	0.000
Macrolides	613 (0.5)	772 (0.4)	404 (0.5)	386 (0.5)	−0.016	−0.003
Sulfamethoxazole-trimethoprim	224 (0.2)	780 (0.4)	192 (0.2)	192 (0.2)	0.042	0.000
Lithium	204 (0.2)	785 (0.4)	182 (0.2)	194 (0.2)	0.046	0.003
Desmopressin	163 (0.1)	310 (0.2)	127 (0.2)	113 (0.1)	0.007	−0.005
Amiodarone	93 (0.1)	222 (0.1)	71 (0.1)	76 (0.1)	0.013	0.002
Education, No.						
Did not complete compulsory education (<9 y)	15 422	20 663	9948	9950	0.078	0.011
Completed compulsory education (9 y)	12 320	20 008	8557	8410		
Upper secondary (2 y)	32 854	50 242	21 901	22 048		
Upper secondary (3 y)	16 486	28 071	11 614	11 617		
College or university (<3 y)	15 135	24 679	10 462	10 412		
College or university (≥3 y)	20 482	35 042	14 309	14 240		
Research education	1712	2829	1193	1230		
Missing	2323	3307	1556	1633		
Annual income, median (IQR), Sk^b^	196 000 (132 900 to 296 200)	215 300 (143 200 to 322 600)	205 700 (137 300 to 310 900)	203 300 (136 900 to 304 100)	0.009	−0.016
Unemployed days, mean	6.0	6.7	6.4	6.5	0.002	0.002

Abbreviations: CCB, calcium channel blocker; COPD, chronic obstructive pulmonary disease; CVD, cerebrovascular disease; IHD, ischemic heart disease; RAS, renin-angiotensin system.

^a^ Refers to model including sodium concentration less than 125 mEq/L (to convert to millimoles per liter, multiply by 1.0), 360 days.

^b^ The current exchange rate: US $1 = Sk9.06.

**Figure 1.**
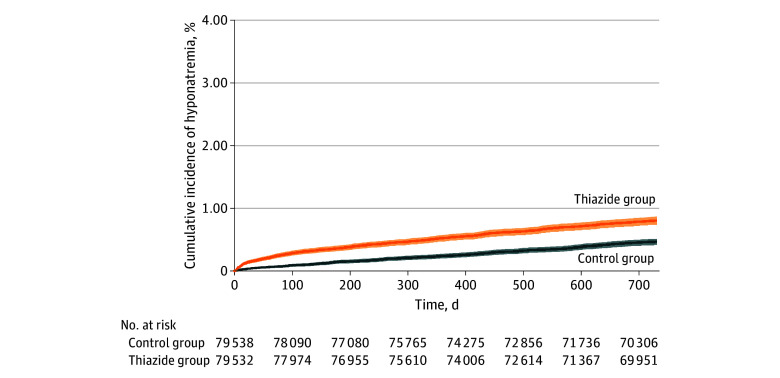
Cumulative Incidences of Profound Hyponatremia Among Individuals Who Initiated Thiazides and Calcium Channel Blockers During 2 Years of Follow-Up Profound hyponatremia was defined as sodium concentration less than 125 mEq/L. Shaded areas indicate 95% CIs. To convert sodium to millimoles per liter, multiply by 1.0.

**Figure 2.**
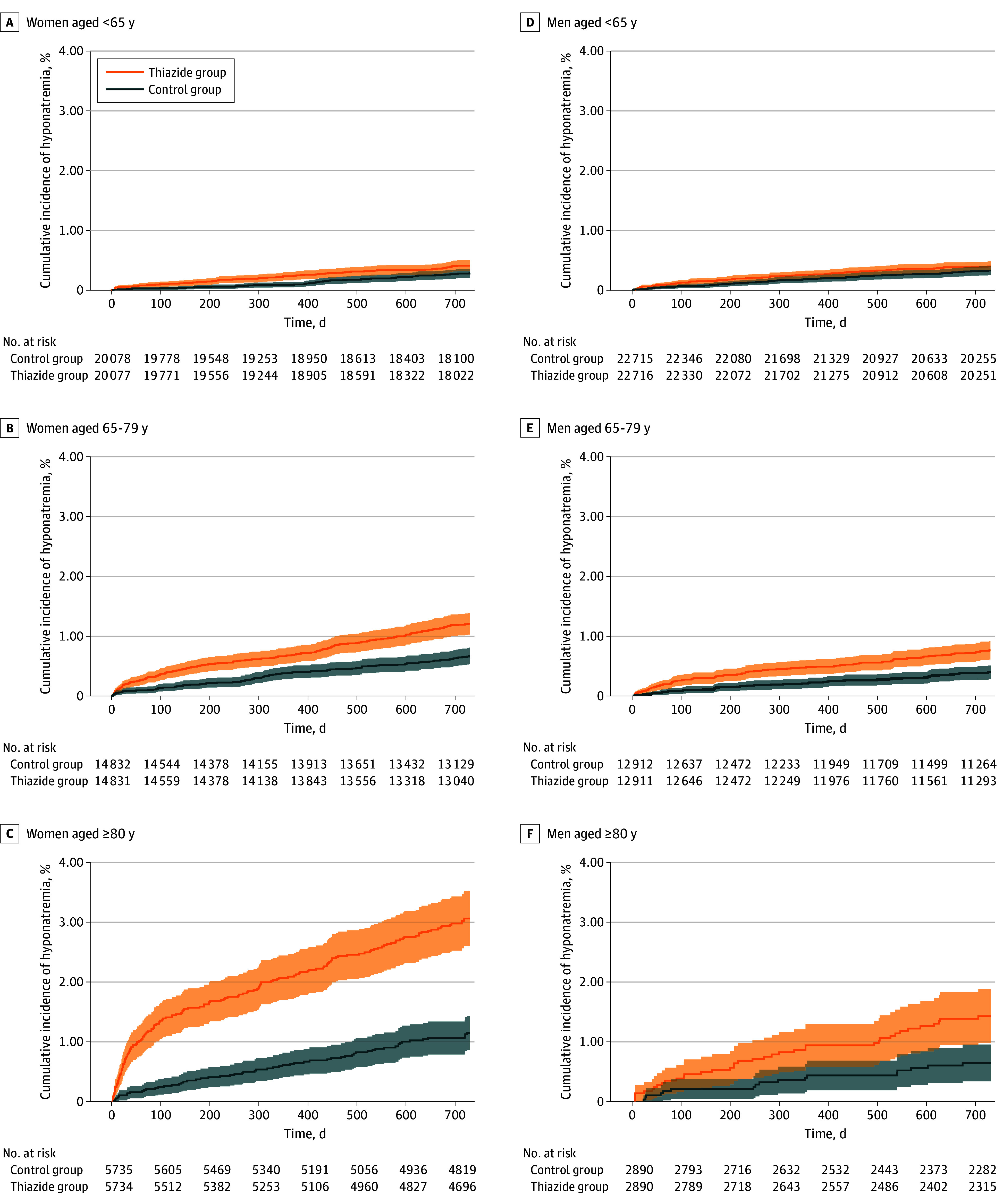
Cumulative Incidences of Profound Hyponatremia Among Individuals Who Initiated Thiazides and Calcium Channel Blockers During 2 Years of Follow-Up Profound hyponatremia was defined as sodium concentration less than 125 mEq/L. Shaded areas indicate 95% CIs. To convert sodium to millimoles per liter, multiply by 1.0.

Figure 1 and Figure 2 illustrate the cumulative incidence of profound hyponatremia among both thiazide and CCB users among all matched individuals and between the sexes according to age. The incidence rates were generally higher during the first months among patients initiating thiazides and then stabilized at a lower level. The cumulative incidences were generally substantially higher among patients receiving thiazides than those receiving CCBs. This increase was particularly marked among older patients and women.

In Table 2, the corresponding associations were quantified in the population as a whole and in subgroups for 14 days and 2 years (730 days). Data regarding the additional time periods 30 days and 120 days are shown in eTable 1 in Supplement 1. During a follow-up time of 2 years, the cumulative incidence of profound hyponatremia among all individuals initiating thiazides was 0.80% (95% CI, 0.74%-0.87%) compared with 0.46% (95% CI, 0.41%-0.51%) for individuals initiating treatment with CCBs (Table 2). The incidence of profound hyponatremia was markedly elevated among women and older individuals. The cumulative incidence was 1.04% (95% CI, 0.94%-1.15%) among women and 0.57% (0.49%-0.64%) among men. The cumulative incidence was 0.40% (95% CI, 0.34%-0.46%) among individuals younger than 65 years, 1.05% (95% CI, 0.93%-1.17%) among those aged 65 to 79 years, and 2.40% (95% CI, 2.07%-2.73%) among those 80 years or older. The corresponding incidence was 3.06% (95% CI, 2.60%-3.52%) for women 80 years or older and 1.43% (95% CI, 0.98%-1.88%) for men 80 years or older. The cumulative incidences associated with CCB use were generally substantially lower than those associated with thiazide use. The cumulative 2-year incidence was 1.11% (95% CI, 0.86%-1.43%) for women 80 years or older initiating CCBs to develop a sodium concentration of less than 125 mEq/L and was 4.78% (95% CI, 4.20%-5.35%) to develop a sodium concentration of less than 130 mEq/L. The cumulative 2-year incidence was 0.29% (95% CI, 0.21%-0.36%) for women younger than 65 years initiating CCBs to develop a sodium concentration of less than 125 mEq/L and was 1.04% (95% CI, 0.90%-1.18%) to develop a sodium concentration of less than 130 mEq/L.

**Table 2.** Incidence and Risk of Profound Hyponatremia Among Patients Receiving Thiazide vs CCB at 14 and 730 Days^a^

Analysis	Cumulative incidence, % (95% CI)		Absolute risk difference, % (95% CI)	No. needed to harm (95% CI)	RR (95% CI)
	Thiazides	CCBs			
**For 14 d**					
All individuals	0.09 (0.07 to 0.12)	0.03 (0.02 to 0.04)	0.07 (0.04 to 0.09)	1472 (1086 to 2283)	3.57 (1.97 to 5.54)
Women	0.12 (0.09 to 0.16)	0.04 (0.02 to 0.06)	0.08 (0.04 to 0.12)	1206 (817 to 2296)	3.00 (1.51 to 4.97)
Men	0.05 (0.03 to 0.07)	0.02 (0.00 to 0.03)	0.03 (0.01 to 0.06)	2963 (1662 to 13 659)	2.86 (0.71 to 6.36)
Age group, y					
<65	0.05 (0.03 to 0.07)	0.01 (0.00 to 0.03)	0.04 (0.01 to 0.06)	2680 (1626 to 7613)	3.67 (0.71 to 8.15)
65-79	0.10 (0.06 to 0.14)	0.05 (0.02 to 0.08)	0.05 (0.00 to 0.10)	1980 (1038 to 21 205)	2.00 (0.89 to 3.74)
≥80	0.21 (0.11 to 0.30)	0.03 (0.00 to 0.07)	0.17 (0.07 to 0.28)	577 (361 to 1401)	6.00 (0.00 to 16.00)
Age group among women, y					
<65	0.05 (0.02 to 0.08)	0.02 (0.00 to 0.03)	0.03 (0.00 to 0.07)	2868 (1427 to ∞)	3.33 (0.00 to 11.40)
65-79	0.11 (0.06 to 0.17)	0.05 (0.02 to 0.13)	0.01 (0.006 to 0.13)	1648 (789 to ∞)	2.12 (0.61 to 4.88)
≥80	0.35 (0.20 to 0.50)	0.07 (0.00 to 0.14)	0.28 (0.11 to 0.45)	358 (224 to 891)	5.00 (0.10 to 12.20)
Age group among men, y					
<65	0.04 (0.02 to 0.07)	0.01 (0.00 to 0.03)	0.03 (0.00 to 0.06)	3243 (1614 to 192 922)	3.33 (0.00 to 11.40)
65-79	0.06 (0.02 to 0.10)	0.02 (0.00 to 0.04)	0.05 (0.00 to 0.09)	2150 (1058 to 117 050)	4.00 (0.00 to 17.3)
≥80	0.14 (0.00 to 0.27)	0	0.14 (0.00 to 0.28)	722 (364 to 33 762)	NC^b^
**For 730 d**					
All	0.80 (0.74 to 0.87)	0.46 (0.41 to 0.51)	0.34 (0.26 to 0.42)	294 (238 to 383)	1.74 (1.52 to 1.98)
Women	1.04 (0.94 to 1.15)	0.57 (0.50 to 0.65)	0.47 (0.35 to 0.60)	213 (168 to 290)	1.82 (1.54 to 2.13)
Men	0.57 (0.49 to 0.64)	0.35 (0.29 to 0.41)	0.21 (0.12 to 0.31)	467 (320 to 857)	1.61 (1.28 to 2.00)
Age group, y					
<65	0.40 (0.34 to 0.46)	0.33 (0.27 to 0.38)	0.08 (−0.01 to 0.16)	1324 (632 to ∞)	1.23 (0.97 to 1.54)
65-79	1.05 (0.93 to 1.17)	0.55 (0.46 to 0.64)	0.50 (0.35 to 0.65)	200 (153 to 286)	1.92 (1.55 to 2.33)
≥80	2.40 (2.07 to 2.73)	0.85 (0.65 to 1.06)	1.55 (1.16 to 1.94)	65 (52 to 86)	2.81 (2.08 to 3.65)
Age group among women, y					
<65	0.41 (0.32 to 0.50)	0.29 (0.21 to 0.36)	0.13 (0.01 to 0.25)	790 (408 to 11 966)	1.44 (1.00 to 2.04)
65-79	1.21 (1.03 to 1.31)	0.67 (0.53 to 0.80)	0.54 (0.031 to 0.77)	186 (131 to 317)	1.81 (1.39 to 2.31)
≥80	3.06 (2.60 to 3.52)	1.11 (0.86 to 1.43)	1.92 (1.38 to 2.46)	53 (41 to 73)	2.67 (1.94 to 3.52)
Age group among men, y					
<65	0.40 (0.32 to 0.48)	0.33 (0.25 to 0.40)	0.07 (−0.04 to 0.18)	1424 (545 to ∞)	1.21 (0.88 to 1.66)
65-79	0.76 (0.61 to 0.92)	0.40 (0.28 to 0.51)	0.37 (0.18 to 0.56)	271 (179 to 558)	1.93 (1.32 to 2.71)
≥80	1.43 (0.98 to 1.88)	0.65 (0.34 to 0.95)	0.78 (0.24 to 1.33)	128 (75 to 424)	2.21 (1.10 to 3.81)

Abbreviations: CCB, calcium channel blocker; NC, not calculated; RR, relative risk.

SI conversion factor: To convert sodium to millimoles per liter, multiply by 1.0.

^a^ Profound hyponatremia was defined as sodium concentration less than 125 mEq/L.

^b^ RR not possible to calculate due to lack of individuals with CCBs in this subgroup.

The absolute risk difference (thiazide vs CCB users) over 2 years of follow-up was 0.34% (95% CI, 0.26%-0.42%) among all individuals (Table 2). The risk difference increased markedly with age and among women. Thus, the risk difference was 0.54% (95% CI, 0.03%-0.77%) for women aged 65 to 79 years and was 1.92% (95% CI, 1.38%-2.46%) for those aged 80 years or older. This corresponded with an NNH of 186 (95% CI, 131-317) for women aged 65 to 79 years and an NNH of 53 (95% CI, 41-73) for women aged 80 years or older. Among women younger than 65 years, the risk difference was 0.13% (95% CI, 0.01%-0.25%), corresponding to an NNH of 790 (95% CI, 408-11 966).

Treatment initiation in the short term was associated with higher RRs compared with treatment initiation in the long term, especially among older adults. Thus, at 14 days, the RR was 3.57 (95% CI, 1.97-5.54) for all individuals compared with 1.74 (95% CI, 1.52-1.98) at 2 years (Table 2). The RR for all individuals 80 years or older was 6.00 (95% CI, 0.00-16.00) at 14 days and 2.81 (95% CI, 2.08-3.65) at 2 years.

In addition, incidences and risks using alternative cutoffs were assessed. The cumulative incidence for a sodium concentration of less than 130 mEq/L was 2.48% (95% CI, 2.37%-2.59%) for all thiazide users (Table 3). The incidences, along with the risk differences, were markedly elevated among women and older individuals. Thus, the cumulative incidence among women 80 years or older was 8.39% (95% CI, 7.65%-9.13%), which contrasted with the cumulative incidence among women younger than 65 years (ie, 1.16% [95% CI, 1.01%-1.31%]). The risk difference was 3.61% (95% CI, 2.68%-4.55%) among women 80 years or older and 0.12% (95% CI, −0.09% to 0.33%) among those younger than 65 years, corresponding to an NNH of 28 (95% CI, 22-38) for women 80 years or older and an NNH of 818 (95% CI, 303-∞) for women younger than 65 years. The risk difference for sodium concentrations less than 130 mEq/L among all individuals 80 years or older was 3.01% (95% CI, 2.32%-3.71%).

**Table 3.** Incidence and Risk of Secondary Hyponatremia Outcomes Among Patients Receiving Thiazide vs CCB at 2-Year Follow-Up

Analysis	Cumulative incidence, % (95% CI)		Absolute risk differences, % (95% CI)	No. needed to harm (95% CI)	RR (95% CI)
	Thiazides	CCBs			
**Sodium <130 mEq/L**					
All individuals	2.48 (2.37 to 2.59)	1.75 (1.65 to 1.84)	0.74 (0.59 to 0.88)	136 (114 to 169)	1.42 (1.33 to 1.52)
Women	2.99 (2.82 to 3.16)	2.19 (2.05 to 2.34)	0.80 (0.58 to 1.02)	126 (98 to 174)	1.36 (1.25 to 1.49)
Men	1.92 (1.78 to 2.06)	1.39 (1.27 to 1.51)	0.53 (0.35 to 0.72)	188 (140 to 287)	1.38 (1.23 to 1.55)
Age group, y					
<65 y	1.22 (1.12 to 1.33)	1.03 (0.93 to 1.13)	0.19 (0.05 to 0.34)	515 (296 to 2006)	1.19 (1.04 to 1.35)
65-79	3.16 (2.95 to 3.37)	2.11 (1.94 to 2.28)	1.05 (0.77 to 1.32)	96 (76 to 130)	1.50 (1.34 to 1.66)
≥80	6.91 (6.35 to 7.46)	3.89 (3.47 to 4.32)	3.01 (2.32 to 3.71)	34 (27 to 44)	1.77 (1.54 to 2.03)
Age group among women, y					
<65	1.16 (1.01 to 1.31)	1.04 (0.90 to 1.18)	0.12 (−0.09 to 0.33)	818 (303 to ∞)	1.12 (0.92 to 1.35)
65-79	3.51 (3.21 to 3.81)	2.29 (2.04 to 2.54)	1.22 (0.83 to 1.61)	82 (62 to 121)	1.53 (1.33 to 1.76)
≥80	8.39 (7.65 to 9.13)	4.78 (4.20 to 5.35)	3.61 (2.68 to 4.55)	28 (22 to 38)	1.76 (1.51 to 2.03)
Age group among men, y					
<65	1.21 (1.06 to 1.36)	1.06 (0.92 to 1.19)	0.16 (−0.04 to 0.35)	647 (283 to ∞)	1.15 (0.96 to 1.37)
65-79	2.61 (2.33 to 2.90)	1.81 (1.57 to 2.05)	0.81 (0.44 to 1.17)	124 (86 to 230)	1.44 (1.21 to 1.71)
≥80	4.04 (3.29 to 4.79)	2.52 (1.92 to 3.13)	1.52 (0.56 to 2.48)	66 (41 to 180)	1.60 (1.16 to 2.15)
**Sodium <135 mEq/L**					
All individuals	8.87 (8.67 to 9.07)	7.49 (7.30 to 7.67)	1.38 (1.11 to 1.66)	73 (61 to 91)	1.18 (1.14 to 1.23)
Women	9.89 (9.60 to 10.19)	8.09 (7.82 to 8.36)	1.80 (1.40 to 2.2)	56 (46 to 72)	1.22 (1.17 to 1.28)
Men	7.51 (7.24 to 7.78)	6.72 (6.47 to 6.98)	0.78 (0.41 to 1.16)	128 (87 to 243)	1.12 (1.06 to 1.18)
Age group, y					
<65	5.52 (5.30 to 5.74)	5.01 (4.80 to 5.22)	0.51 (0.20 to 0.81)	199 (124 to 503)	1.10 (1.04 to 1.17)
65-79	10.48 (10.11 to 10.84)	8.55 (8.22 to 8.89)	1.92 (1.42 to 2.42)	52 (42 to 71)	1.22 (1.16 to 1.29)
≥80	20.45 (19.57 to (21.32)	15.84 (15.03 to 16.63)	4.61 (3.43 to 5.8)	22 (18 to 30)	1.29 (1.21 to 1.38)
Age group among women, y					
<65	5.77 (5.44 to 6.10)	4.94 (4.63 to 5.24)	0.83 (0.38 to 1.28)	120 (78 to 261)	1.17 (1.07 to 1.27)
65-79	11.16 (10.64 to 11.68)	8.87 (8.40 to 9.34)	2.29 (1.59 to 2.99)	44 (34 to 63)	1.26 (1.17 to 1.35)
≥80	22.60 (21.48 to 23.71)	16.10 (15.11 to 17.08)	6.50 (5.01 to 7.99)	16 (13 to 20)	1.40 (1.30 to 1.52)
Age group among men, y					
<65	5.21 (4.92 to 5.51)	5.09 (4.79 to 5.38)	0.13 (−0.29 to 0.54)	788 (185 to ∞)	1.02 (0.95 to 1.11)
65-79	9.45 (8.93 to 9.97)	8.23 (7.74 to 8.72)	1.22 (0.51 to 1.93)	82 (52 to 196)	1.15 (1.06 to 1.24)
≥80	16.86 (15.43 to 18.27)	13.86 (12.53 to 15.17)	3.00 (1.06 to 4.94)	34 (21 to 95)	1.22 (1.07 to 1.38)

Abbreviations: CCB, calcium channel blocker; RR, relative risk.

SI conversion factor: To convert sodium to millimoles per liter, multiply by 1.0.

The cumulative incidence of a sodium concentration of less than 135 mmEq/L for the population as a whole was 8.87% (95% CI, 8.67%-9.07%) for the thiazide group and 7.49% (95% CI, 7.30%-7.67%) for individuals initiating CCBs (Table 3). Again, the incidences, along with the risk differences, were markedly elevated among women and older individuals. The incidence of a sodium concentration of less than 135 mmEq/L in the thiazide group was 22.60% (95% CI, 21.48%-23.71%) among women 80 years or older compared with 5.77% (95% CI, 5.44%-6.10%) among women younger than 65 years. The risk difference was 6.50% (95% CI, 5.01%-7.99%) among women 80 years or older and 0.83% (95% CI, 0.38%-1.28%) among women younger than 65 years, corresponding to an NNH of 16 (95% CI, 13-20) for women 80 years or older and an NNH of 120 (95% CI, 78-261) for women younger than 65 years.

To illustrate the total number of sodium measurements between thiazides and CCBs, the frequencies were compared in the overall population and in subgroups. Sodium testing tended to be more frequent among patients initiating CCBs (eTable 3 in Supplement 1). In the per-protocol sensitivity analysis, the absolute 2-year risks of profound hyponatremia, in particular among thiazide users, decreased substantially along with the absolute risk differences (eTable 4 in Supplement 1).

An analysis of the association of initiation of thiazides and CCBs with all-cause mortality was performed as a negative control. The results were similar, although mortality in the CCB group was slightly higher (eTable 5 in Supplement 1). Cause-specific hazard modeling of death with hyponatremia treated as a censoring event indicated lower mortality in the thiazide group compared with the control group (risk ratios ranging from 0.86 to 0.90 and absolute risk differences ranging from −0.26% to −0.82% over the full 2-year period at different degrees of hyponatremia).

## Discussion

In the present study, 79 540 individuals starting thiazide treatment were propensity score matched with 79 540 individuals receiving CCBs and compared regarding subsequent hyponatremia. The cumulative incidence of profound hyponatremia (sodium <125 mEq/L) was 0.80% for thiazides and 0.46% for CCBs during the first 2 years of treatment. The occurrence was markedly higher among women and older individuals. Thus, among women 80 years or older, the NNH was 53 for a sodium concentration less than 125 mEq/L, was 28 for a sodium concentration less than 130 mEq/L, and was 16 for a sodium concentration less than 135 mEq/L. This was in marked contrast with women younger than 65 years, for whom the corresponding NNH was 719 for a sodium concentration less than 125 mEq/L, 818 for a sodium concentration less than 130 mEq/L, and 120 for a sodium concentration less than 135 mEq/L.

The higher risk among women and older individuals was to some extent associated with a higher background risk. Thus, as illustrated by Figure 2, older individuals and women initiating CCBs, drugs not linked with hyponatremia to the same degree, were associated with a markedly higher occurrence of hyponatremia. For women 80 years or older initiating CCBs, the cumulative 2-year incidence was 1.11% to develop sodium concentrations less than 125 mEq/L and 4.78% to develop sodium concentrations less than 130 mEq/L. For women younger than 65 years initiating CCBs, the cumulative 2-year incidence was 0.29% to develop sodium concentrations less than 125 mEq/L and 1.04% to develop sodium concentrations less than 130 mEq/L. Nevertheless, the risk specifically associated with thiazides (the absolute risk difference) was considerable, reflected by a relatively low NNH.

In a clinical context, to adequately communicate and mitigate any adverse effects, it is essential to picture the overall anticipated risk. Predicting the risk of hyponatremia, regardless of baseline risk, may therefore be equally important when initiating thiazide diuretics. The results of the present study demonstrate that the risk of hyponatremia, irrespective of the cutoff used, is markedly elevated among older individuals, particularly women. For instance, among women 80 years or older, 1 in every 33 women who initiated thiazide treatment developed profound hyponatremia within 2 years of baseline. Using the cutoff serum sodium concentration of less than 130 mEq/L, 1 in every 12 women aged 80 years or older developed hyponatremia, and using a cutoff serum sodium concentration of less than 135 mEq/L, 1 in every 5 women aged 80 years or older developed hyponatremia. In contrast, among women younger than 65 years, 1 in every 244 developed profound hyponatremia within 2 years of baseline, 1 in every 87 developed a serum sodium concentration of less than 130 mEq/L, and 1 in every 18 developed a serum sodium concentration of less than 135 mEq/L.

The association between hyponatremia and female sex and age, respectively, is in line with previous evidence. Based on a meta-analysis, Barber et al^3^ found that the mean age for patients presenting with thiazide-induced hyponatremia was 75 years (based on 36 studies and 2840 individuals). The proportion of women was 79% (based on 43 studies and 3269 patients). In a Danish study,^10^ target trials were emulated for individuals 40 years or older comparing thiazides and other first-line antihypertensive drugs. In contrast with these studies, the design of the Danish study allowed for the investigation of absolute risks. Individuals initiating BFT (n = 37 786) had additional 2-year cumulative incidences of sodium concentrations less than 130 mEq/L of 3.8%, and those initiating HCT (n = 11 943) had 2-year cumulative incidences of sodium concentrations less than 130 mEq/L of 3.5% compared with individuals starting CCBs or renin-angiotensin system inhibitors alone, figures slightly higher than in the present study (2.48%). Some part of this discrepancy may be based on the fact that Andersson et al^10^ excluded individuals younger than 40 years. Although the 95% CIs overlapped, the risk difference for sodium concentrations less than 130 mEq/L among individuals 80 years or older in the study by Andersson et al^10^ was 4.80 (95% CI, 3.36-6.25), slightly higher than that in the present study (3.01% [95% CI, 2.32%-3.71%]). We speculate that the underlying reasons for this imbalance may be explained by differences between health care and the measurement of sodium concentrations among patients receiving hypertensive treatment. Another potential source of the imbalance could be differences in the covariates used, resulting in variable confounding. Furthermore, Andersson et al^10^ performed a subgroup analysis dividing the population according to sex in which no difference was seen. This finding contrasts with the present study, which showed an elevated risk for women in the population as a whole. Further dividing the sexes into different age groups, it was evident that this difference was largely seen among older women, who had a markedly increased risk compared with men. The risk ratios were similar across the different populations, suggesting that the differences in absolute risk were associated with baseline differences rather than a true modification of thiazide’s association with hyponatremia.

Our finding of the highest rate of hyponatremia in the first months after thiazide initiation is in line with previous observational studies.^10,15^ Furthermore, the intention-to-treat design assumed that a first filled prescription equaled drug use throughout the study period and might have underestimated the true occurrence of hyponatremia compared with a per-protocol analysis. A per-protocol analysis on the 2-year risk of profound hyponatremia showed that the absolute risk for thiazides decreased substantially along with the risk differences. These results are consistent with a substantial risk for hyponatremia, disproportionately seen in the thiazide group, particularly shortly after initiation, associated with premature discontinuation and exclusion from the per-protocol dataset. However, small risk differences remained, reflecting a risk increase that is known to remain elevated even long after initiation.^6^

To address potential residual confounding, we included all-cause mortality as a negative control. The results between the groups were similar, although mortality was slightly higher among individuals initiating CCBs. This tendency was more pronounced during longer follow-up. The analysis suggests that individuals initiating thiazides may be healthier. The finding may indicate some degree of bias underestimating the difference between thiazides and CCBs with regard to the occurrence of hyponatremia.

### Strengths and Limitations

This study has some strengths. The main strength is the use of the SSC, a research register specifically aimed at exploring hyponatremia. The SSC was sufficiently large to permit conclusive results not only regarding the population as a whole but also in subgroups, in particular among sex-specific age groups. The SSC includes a wide range of variables that helped us to achieve balanced groups in the propensity score matching.

However, this study also has some limitations. Because it is an observational study, we cannot exclude some degree of residual confounding (eg, additional underlying diseases). Theoretically, censoring at death could have introduced bias if death was informative of future hyponatremia risk. Cause-specific hazards modeling of death indicated somewhat lower mortality rates in the thiazide group, introducing a risk that more severely ill individuals with a high underlying risk of hyponatremia remained at risk for longer in the thiazide group. However, the lower death rates should not be interpreted as a true association of thiazide treatment with mortality, but rather as a consequence of differential depletion of the risk set due to competing risks. In addition, the absolute risk differences were very small (<1%), making it unlikely that informative censoring due to competing risks substantially biased the results. Finally, although the CCB group was chosen as a null reference with regard to the risk of hyponatremia, a weaker association cannot be excluded, which may lead to an underestimation of the risk difference and NNH.^16^

## Conclusions

Although recognizing the general risk of hyponatremia, the recently published *2025 AHA/ACC/AANP/AAPA/ABC/ACCP/ACPM/AGS/AMA/ASPC/NMA/PCNA/SGIM Guideline for the Prevention, Detection, Evaluation, and Management of High Blood Pressure in Adults* unfortunately does not stratify the risk regarding specific patient groups.^17^ Thus, the results of the present cohort study have important clinical implications. For older individuals, especially women, thiazide treatment was associated with a substantial risk of hyponatremia that should be considered in the risk-benefit assessment and incentivize the prescriber to choose an alternative antihypertensive treatment. If thiazides are prescribed, the patient should be carefully informed about symptoms such as fatigue, confusion, and balance disturbances, which may indicate developing hyponatremia and should prompt seeking immediate medical attention and subsequent sodium concentration analysis. Regular serum sodium monitoring should be considered. The findings of this study suggest that this risk is relatively higher during the first weeks or months after starting thiazides but remains elevated even in the long term. In contrast, for younger individuals, this risk is likely to be negligible.

## Notes

Supplement 1. **eTable 1.** Definition of Exposures Under Study and Potential Confounders Variables Used for the Propensity Score Matching **eTable 2.** Cumulative Incidences for Thiazides and Calcium Channel Blockers, Absolute Risk Differences and Hazard Ratios of Hyponatremia (Sodium Concentration <125 mmol/L) for Time Periods 30 and 90 Days Overall and by Subgroups **eTable 3.** Distribution of Sodium Measurements for Thiazides and Calcium Channel Blockers, During 14, 30, 90, 730 Days Follow-Up Overall and by Subgroups **eTable 4.** Cumulative Incidences for Thiazides and CCBs, Absolute Risk Differences and HRs of Hyponatremia for Secondary Outcomes (Sodium <125, <135 and <130 mmol/L Respectively) for 2 Years Follow-Up Overall Using Per Protocol Analysis **eTable 5.** Cumulative Incidences, Absolute Risk Differences, Number Needed to Harm and Risk Ratios for All-Cause Mortality After Initiation of Thiazides and Calcium Channel Blockers at 14, 30, 90, 730 Days Follow-Up in the Overall Population and by Subgroups **eFigure.** Study Flow Diagram of the Study Population

Supplement 2.
Data Sharing Statement
