# Error in Figures

**DOI:** 10.1001/jamanetworkopen.2026.25217

**Published:** 2026-06-30

In the Original Investigation titled “Thiazides and Risk of Hyponatremia by Age and Sex,”^1^ published April 2, 2026, the values in the y-axes in Figures 1 and 2 were incorrect. Instead of cumulative incidences being expressed as proportions (unitless values from 0 to 1), they should be expressed as percentages. This article has been corrected.^1^
