# Supplementary material for: Thiazides and Risk of Hyponatremia by Age and Sex
Source: JAMA Netw Open. 2026 Apr 2;9(4):e264642. doi: 10.1001/jamanetworkopen.2026.4642 (PMC13047463; doi:10.1001/jamanetworkopen.2026.4642)
Supplement: Supplement 1. — eTable 1. Definition of Exposures Under Study and Potential Confounders Variables Used for the Propensity Score Matching eTable 2. Cumulative Incidences for Thiazides and Calcium Channel Blockers, Absolute Risk Differences and Hazard Ratios of Hyponatremia (Sodium Concentration <125 mmol/L) for Time Periods 30 and 90 Days Overall and by Subgroups eTable 3. Distribution of Sodium Measurements for Thiazides and Calcium Channel Blockers, During 14, 30, 90, 730 Days Follow-Up Overall and by Subgroups eTable 4. Cumulative Incidences for Thiazides and CCBs, Absolute Risk Differences and HRs of Hyponatremia for Secondary Outcomes (Sodium <125, <135 and <130 mmol/L Respectively) for 2 Years Follow-Up Overall Using Per Protocol Analysis eTable 5. Cumulative Incidences, Absolute Risk Differences, Number Needed to Harm and Risk Ratios for All-Cause Mortality After Initiation of Thiazides and Calcium Channel Blockers at 14, 30, 90, 730 Days Follow-Up in the Overall Population and by Subgroups eFigure. Study Flow Diagram of the Study Population [file jamanetwopen-e264642-s001.pdf]

Supplementary Online Content

Bergh Fahlén C, Falhammar H, Skov J, Lindh JD, Mannheimer B. Thiazides and risk of hyponatremia by age and sex. *JAMA Netw Open.* 2026;9(4):e264642. doi:10.1001/jamanetworkopen.2026.4642

eTable 1. Definition of Exposures Under Study and Potential Confounders Variables Used for the Propensity Score Matching

eTable 2. Cumulative Incidences for Thiazides and Calcium Channel Blockers, Absolute Risk Differences and Hazard Ratios of Hyponatremia (Sodium Concentration <125 mmol/L) for Time Periods 30 and 90 Days Overall and by Subgroups

eTable 3. Distribution of Sodium Measurements for Thiazides and Calcium Channel Blockers, During 14, 30, 90, 730 Days Follow-Up Overall and by Subgroups

eTable 4. Cumulative Incidences for Thiazides and CCBs, Absolute Risk Differences and HRs of Hyponatremia for Secondary Outcomes (Sodium <125, <135 and <130 mmol/L Respectively) for 2 Years Follow-Up Overall Using Per Protocol Analysis

eTable 5. Cumulative Incidences, Absolute Risk Differences, Number Needed to Harm and Risk Ratios for All-Cause Mortality After Initiation of Thiazides and Calcium Channel Blockers at 14, 30, 90, 730 Days Follow-Up in the Overall Population and by Subgroups

eFigure. Study Flow Diagram of the Study Population

This supplementary material has been provided by the authors to give readers additional information about their work.

© 2026 Bergh Fahlén C et al. *JAMA Network Open.*

eTable 1. Definition of Exposures Under Study and Potential Confounders Variables Used for the Propensity Score Matching

**Exposures under study**	
Thiazides	C03AA, C03EA01, C09BA or C09DA
Calcium channel blockers	C08C, C09BB or C09DB
**Potential confounders**	
Comorbidities	*ICD[1]/ATC[2]/Procedure-codes*
Malignancy	C
Diabetes mellitus	ICD E10.0, E10.1, E10.9, E11.0, E11.1, E11.9 / ATC A10
IHD[3] ≥90 days	I20-I25
Atrial fibrillation	I48
CVD[4] ≥ 90 days	I60-64, I69
Chronic pulmonary disease other	J40-J43, J45-J47, J60-J67, J68.4, J70.1, J70.3, J84.1, J92.0, J96.1, J98.2, J98.3
COPD[5]	ICD J44 / ATC R03
Alcohol/drug abuse	E244, F10-F16, G312, G621, G721, I426, K292, K70, K860, O354, P043, Q860, T51, Y90-91, Z714, F18-F19, T40, T43, R781-R785, Z722
Congestive heart failure	I11.0, I13.0, I13.2, I50
Hypothyroidism	E03, E06.3
CVD[4] <90 days	I60-64
Rheumatic disease	M05, M06, M08, M09, M30, M31, M32, M33, M34, M35, M36, D86
Liver disease	K65, K70-K77, I982, Z944, D684C, procedure codes JJB, JJC
**Renal disease**	**I12, I13, N17-19, procedure codes DR016, DR024, KAS00, KAS10, KAS20 OR eGFR<45 (mL/min/1,73m^2)**
Smoking	F17, Z71.6, Z72.0
Pancreatic disease	K85, K860-1
Dementia	F00.0, F01, F02, F03, F05, G30, G31.1, G31.8
IHD[3] <90 days	I20-24
Inflammatory bowel disease	K51, K50
Pneumonia <90 days	J18
Gastroenteritis <90 days	A0, J108A, J118B, K52
Adrenal failure	E27.1, E27.2, E27.3, E27.4, E25
Acute pulmonary embolism <90 days	I26
Sepsis <90 days	A41
Cerebral infection <90 days	G00-G07
Malnutrition	E43.9, E41.9
Medications	
RAS inhibitors[6]	C09
β-blockers	C07
Lipid lowering agents	C10
Proton pump inhibitors	A02BC
Antidepressants	N06A

© 2026 Bergh Fahlén C et al. *JAMA Network Open.*

Opioids	N02A
Furosemide	C03CA01
Antiepileptics	N03A
Quinolones	J01MA
Antipsychotics	N05A excluding N05AN
Macrolides	J01FA
Sulfamethoxazole and trimethoprim	J01EE
Lithium	N05AN
Desmopressin	H01BA02
Amiodarone	C01BD01
**Education and income**	
Education<br>Categorial variable in seven strata	*; 1; 2; 3; 4; 5; 6; 7 : 2323; 15 422; 12 320; 32 854; 16 486; 15 135; 20 482; 1712
Annual income, median (IQR)(SEK)	196 000 (132 900–296 200)
**Matching other**	according to calender year of treatment initiation

[1]ICD-code; International classification of diseases code
[2]ATC-code; Anatomical Therapeutic Chemical classification code
[3]IHD; Ischemic heart disease
[4]CVD; Cerebrovascular disease
[5]COPD; Chronic obstructive pulmonary disease
[6]RAS inhibitors; Renin-angiotensin system inhibitors

© 2026 Bergh Fahlén C et al. *JAMA Network Open.*

eTable 2. Cumulative Incidences for Thiazides and Calcium Channel Blockers, Absolute Risk Differences and Hazard Ratios of Hyponatremia (Sodium Concentration <125 mmol/L) for Time Periods 30 and 90 Days Overall and by Subgroups

**Analysis**	**Cumulative incidence thiazides (95% CI[1])**	**Cumulative incidence CCBs[2] (95% CI)**	**Absolute risk difference (95% CI[1])**	**Number Needed to Harm (95% CI[1])**	**RR[3] (95% CI)**
30 days					
All	0.15 (0.13–0.18)	0.04 (0.03–0.06)	0.11 (0.08–0.14)	892 (700-1229)	3.70 (2.36–5.26)
Women	0.20 (0.16–0.25)	0.07 (0.04–0.09)	0.14 (0.09–0.19)	732 (535-1155)	3.07 (1.84–4.59)
Men	0.07 (0.05–0.10)	0.02 (0.00–0.04)	0.05 (0.02-0.08)	1925 (1212-4666)	3.50 (1.03–7.06)
Age <65	0.07 (0.04–0.09)	0.02 (0.00–0.03)	0.05 (0.02–0.08)	2041 (1315-4554)	4.00 (1.00–8.23)
Age 65–79	0.16 (0.11–0.20)	0.07 (0.04–0.10)	0.09 (0.03–0.14)	1154 (702-3226)	2.26 (1.18–3.78)
Age ≥80	0.45 (0.31–0.59)	0.07 (0.01–0.13)	0.38 (0.23–0.54)	262 (187-434)	6.50 (1.27–13.10)
Women <65	0.05 (0.002-0.09)	0.01 (0.00-0.03)	0.04 (00--0.08)	2509 (1309-23801)	3.67 (0.00-11.80)
Women 65-79	0.20 (0.13-0.27)	0.09 (0.04-0.14)	0.12 (0.03-0.20)	872 (496-3565)	2.31 (0.99-4.29)
Women ≥80C	0.74 (0.51–0.96)	0.10 (0.02–0.19)	0.63 0.39–0.87)	159 (115-254)	7.01 (1.37–14.00)
Men <65	0.06 (0.00-0.09)	0.02 (0.00-0.04)	0.04 (0.00-0.07)	2522 (1293-49559)	2.80 (0.33-7.40)
Men 65-79	0.09 (0.00-0.15)	0.03 (0.00-0.06)	0.06 (0.00-0.12)	1612 (813-79612)	3.00 (0.06-8.81)
Men ≥80C	0.17 (0.02-0.33)	0.10 (0.00-0.22)	0.07 (-0.12-0.26)	1451 (382-∞)	1.66 (0.00-15.30)
90 days					
All	0.27 (0.23–0.30)	0.08 (0.06–0.10)	0.19 (0.15–0.23)	535 (438-685)	3.31 (2.43–4.30)
Women	0.36 (0.30–0.41)	0.11 (0.08–0.14)	0.25 (0.18–0.31)	409 (322-559)	3.22 (2.21–4.40)
Men	0.17 (0.13–0.21)	0.06 (0.04–0.09)	0.11 (0.06–0.16)	935 (644-1701)	2.7 (1.55–4.19)
<65	0.12 (0.08–0.15)	0.05 (0.03–0.07)	0.07 (0.03–0.11)	1474 (939-3424)	2.38 (1.29–3.85)
65–79	0.30 (0.24–0.37)	0.11 (0.08–0.16)	0.19 (0.11-0.26)	541 (382-925)	2.55 (1.61-3.72)
≥80	0.87 (0.68–1.07)	0.18 (0.09–0.26)	0.70 (0.48–0.92)	144 (109-207)	4.99 (2.40-8.09)
Women <65	0.10 (0.05–0.14)	0.03 (0.01–0.05)	0.07 (0.02–0.11)	1538 (877-6224)	3.17 (0.61–7.33)
Women 65-79	0.32 (0.23–0.42)	0.11 (0.06–0.16)	0.22 (0.11–0.32)	462 (310-904)	3.00 (1.46–5.04)
Women ≥80	1.28 (1.00–1.58)	0.23 (0.10–0.35)	1.06 (0.74–1.37)	95 (73-136)	5.62 (2.50–9.32)
Men <65	0.12 (0.07–0.16)	0.06 (0.03–0.09)	0.05 (0.00–0.11)	1885 (927-∞)	1.86 (0.83–3.53)
Men 65-79	0.25 (0.16–0.34)	0.09 (0.04–0.14)	0.16 (0.06-0.26)	611 (379-1573)	2.91 (1.13-5.45)
Men ≥80	0.39 (0.16–0.61)	0.21 (0.04–0.38)	0.18 (-0.11-0.46)	573 (218-925)	1.83 (0.34–5.32)

[1] CI, Confidence Interval.
[2] CCBs, Calcium Channel Blockers
[3] HR, Hazard ratio

© 2026 Bergh Fahlén C et al. *JAMA Network Open.*

eTable 3. Distribution of Sodium Measurements for Thiazides and Calcium Channel Blockers, During 14, 30, 90, 730 Days Follow-Up Overall and by Subgroups

Analysis	N, median (IQR[1]) sodium measurements for thiazides	Median (IQR) sodium measurements for CCBs	p-value
14 days			
All individuals	5682, 0 (0-0)	6361 (0 (0-0)	<0.01
Women	3032, 0 (0-0)	3510, 0 (0-0)	<0.01
Men	2544, 0 (0-0)	2954, 0 (0-0)	<0.01
<65	2751, 0 (0-0)	3366, 0 (0-0)	<0.01
65–79	2045, 0 (0-0)	2147, 0 (0-0)	0.10
≥80	820, 0 (0-0)	813, 0 (0-0)	0.80
Women <65	1361, 0 (0-0)	1719, 0 (0-0)	<0.01
Women 65-79	1146, 0 (0-0)	1119, 0 (0-0)	0.56
Women ≥80	546, 0 (0-0)	561, 0 (0-0)	0.67
Men <65	1362, 0 (0-0)	1674, 0 (0-0)	<0.01
Men 65-79	940, 0 (0-0)	965, 0 (0-0)	0.56
Men ≥80	280, 0 (0-0)	243, 0 (0-0)	0.09
30 days			
All individuals	10 055, 0 (0-0)	10 038, 0.0 (0-0)	0.87
Women	5342, 0 (0.0-0)	5507, 0 (0-0)	0.06
Men	4585, 0 (0-0)	4676, 0 (0-0)	0.22
<65	4935, 0 (0-0)	5183, 0 (0-0)	<0.01
65–79	3667, 0 (0-0)	3448, 0 (0-0)	<0.01
≥80	1430, 0 (0-0)	1373, 0 (0-0)	0.21
Women <65	2 421, 0 (0-0)	2 603, 0 (0-0)	<0.01
Women 65-79	2 020, 0 (0-0)	1 807, 0 (0-0)	<0.01
Women ≥80	970, 0 (0-0)	888, 0 (0-0)	0.04
Men <65	2485, 0 (0-0)	2579, 0 (0-0)	0.13
Men 65-79	1668, 0 (0-0)	1533, 0 (0-0)	0.02
Men ≥80	478, 0 (0-0)	445, 0 (0-0)	0.24
90 days			
All individuals	17 307, 0 (0-0)	17 445, 0.0 (0-0)	0.08
Women	9181, 0 (0-0)	9266, 0 (0-0)	0.18

© 2026 Bergh Fahlén C et al. *JAMA Network Open.*

Men	8010, 0 (0-0)	8182, 0 (0-0)	0.04
<65	8615, 0 (0-0)	8803, 0 (0-0)	0.02
65–79	6206, 0 (0-0)	6134, 0 (0-0)	0.68
≥80	2475, 0 (0-1)	2427, 0 (0-1)	0.32
Women <65	4171, 0 (0-0)	4304, 0 (0-0)	0.03
Women 65-79	3362, 0 (0-0)	3243, 0 (0-0)	0.22
Women ≥80	1655, 0 (0-1)	1572, 0 (0-1)	0.06
Men <65	4365, 0 (0-0)	4491, 0 (0-0)	0.05
Men 65-79	2877, 0 (0-0)	2810, 0 (0-0)	0.48
Men ≥80	832, 0 (0-1)	829, 0 (0-1)	0.89
730 days			
All individuals	43 669, 1 (0-2)	44 362, 1 (0-2)	<0.01
Women	23149, 1 (0-2)	23264, 1 (0-2)	0.05
Men	20392, 1 (0-2)	20952, 1 (0-2)	<0.01
<65	21947, 1 (0-2)	22242, 1 (0-2)	<0.01
65–79	15758, 1 (0-3)	15858, 1 (0-3)	0.27
≥80	5894, 2 (0-5)	5960, 2 (0-5)	0.59
Women <65	10538, 1 (0-2)	10581, 1 (0-2)	0.26
Women 65-79	8525, 1 (0-3)	8546, 1 (0-3)	0.69
Women ≥80	3900, 2 (0-5)	3951, 2 (0-5)	0.56
Men <65	11257, 0 (0-2)	11567, 0 (0-2)	<0.01
Men 65-79	7213, 1 (0-3)	7362, 1 (0-3)	0.02
Men ≥80	1975, 2 (0-5)	1969, 2 (0-5)	0.95

[1]IQR

© 2026 Bergh Fahlén C et al. *JAMA Network Open.*

eTable 4. Cumulative Incidences for Thiazides and CCBs[2], Absolute Risk Differences and HRs of Hyponatremia for Secondary Outcomes (Sodium <125, <135 and <130 mmol/L Respectively) for 2 Years Follow-Up Overall Using Per Protocol Analysis

**Analysis**	**Cumulative incidence thiazides % (95 % CI[1])**	**Cumulative incidence CCBs[2] % (95 % CI[1])**	**Absolute risk differences % (95% CI[1])**	**Number Needed to Harm (95% CI[1])**	**RR[3] (95% CI[1])**
Sodium <125 mmol/L	0.20 (0.12-0.29)	0.16 (0.09-0.23)	0.05 (-0.06-0.16)	2055 (629-∞)	0.63 (0.42-0.99)
Sodium <130 mmol/L	1.08 (0.88-1.27)	0.84 (0.68-1.06)	0.23 (0.00-0.49)	429 (205-∞)	1.28 (0.97-1.67)
Sodium <135 mmol/L	5.39 (4.97-5.82)	4.86 (4.48-5.25)	0.53 (-0.04-0.01)	189 (90.7-∞)	1.11 (0.99-1.24)

[1]Confidence Intervall
[2]Calcium Channel Blockers
[3]Risk Ratios

© 2026 Bergh Fahlén C et al. *JAMA Network Open.*

eTable 5. Cumulative Incidences, Absolute Risk Differences, Number Needed to Harm and Risk Ratios for All-Cause Mortality After Initiation of Thiazides and Calcium Channel Blockers at 14, 30, 90, 730 Days Follow-Up in the Overall Population and by Subgroups

**Analysis**	**Cumulative incidence thiazides % (95 % CI[1])**	**Cumulative incidence CCBs[2] % (95 % CI[1])**	**Absolute risk differences % (95% CI[1])**	**Number Needed to Harm (95% CI)**	**RR[3] (95% CI[1])**
14 days					
All individuals	0.05 (0.03-0.06)	0.04 (0.03-0.055)	0.01 (-0.01-0.03)	19862 (3943-∞)	1.13 (0.68-1.85)
Women	0.03 (0.02-0.05)	0.05 (0.03-0.07)	-0.01 (-0.04-0.02)	-8205 (6551- -2523)	0.74 (0.36-1.69)
Men	0.06 (0.04-0.08)	0.02 (0.01-0.04)	0.04 (0.01-0.07)	2569 (1487-9419)	2.87 (0.84-6.05)
<65	0.01 (0.00 - 0.03)	0.02 (0.01-0.03)	0.00 (-0.02-0.01)	-21426 (8039- -4593)	0.75 (0.20-4.08)
65–79	0.05 (0.02-0.08)	0.05 (0.02-0.07)	0.00 (-0.03-0.04)	27587 (2478-∞)	1.08 (0.44-2.54)
≥80	0.20 (0.10-0.29)	0.23 (0.13-0.33)	-0.04 (-0.17-0.10)	-2887 (972- -581)	0.85 (0.42-1.78)
Women <65	0.01 (0.00-0.02)	0.02 (0.00-0.03)	-0.01 (-0.02- -0.01)	-10034 (13038 - -3594)	0.33 (0.00-∞)
Women 65-79	0.03 (0.00-0.01)	0.03 (0.00-0.05)	0.01 (-0.03-0.05)	14822 (2155-∞)	1.25 (0.024-11.30)
Women ≥80	0.16 (0.05-0.26)	0.24 (0.12-0.37)	-0.09 (-0.25-0.08)	-1147 (1305- -399)	0.64 (0.26-2.01)
Men <65	0.01 (0.00-0.03)	0.02 (0.00-0.04)	0.00 (-0.03-0.02)	-22697 (5768- -3670)	0.75 (0.014-∞)
Men 65-79	0.07 (0.02-0.12)	0.02 (0.00-0.05)	0.05 (-0.05-0.01)	2150 (1009-∞)	3.00 (0.00-11.00)
Men ≥80	0.35 (0.13-0.56)	0.17 (0.02-0.32)	0.17 (-0.09-0.44)	579 (230-∞)	2.00 (0.23-6.33)
30 days					
All individuals	0.13 (0.10-0.15)	0.10 (0.08-0.12)	0.03 (0.01-0.06)	3968 (1710-∞)	1.25 (0.92-1.67)
Women	0.11 (0.08-0.14)	0.10 (0.07-0.13)	0.01 (-0.04- 0.05)	10215 (1847-∞)	1.10 (0.70-1.70)
Men	0.14 (0.10-0.18)	0.10 (0.07-0.13)	0.04 (0.01-0.09)	2408 (1107-∞)	1.42 (0.91-2.15)
<65	0.04 (0.02-0.06)	0.04 (0.02-0.06)	-0.01 (-0.03-0.02)	-14273 (4981- -2934)	0.84 (0.41-1.82)
65–79	0.09 (0.06-0.13)	0.09 (0.05-0.12)	0.01 (-0.04-0.06)	1384 (1747-∞)	1.08 (0.59-1.95)
≥80	0.65 (0.48-0.82)	0.47 (0.33-0.62)	0.17 (-0.05-0.40)	577 (253-∞)	1.37 (0.89-2.04)
Women <65	0.03 (0.01-0.05)	0.02 (0.00-0.03)	0.02 (-0.01-0.04)	6658 (2254-∞)	2.00 (0.00-11.90)
Women 65-79	0.05 (0.01-0.08)	0.05 (0.02-0.09)	0.01 (-0.06-0.04)	-14708 (2250- -1723)	0.87 (0.24-3.72)
Women ≥80	0.56 (0.37-0.75)	0.52 (0.34-0.71)	0.04 (-0.23-0.30)	2833 (329-∞)	1.07 (0.63-1.80)
Men <65	0.04 (0.02-0.07)	0.05 (0.02-0.08)	-0.004 (-0.04-0.04)	-22733 (2841- -2273)	0.91 (0.33-2.66)
Men 65-79	0.13 (0.07-0.19)	0.09 (0.04-0.15)	0.04 (-0.04-0.12)	2583 (830-∞)	1.42 (0.56-3.10)
Men ≥80	0.87 (0.53-1.20)	0.52 (0.26-0.78)	0.35 (-0.08-0.77)	289 (130-∞)	1.67 (0.77-3.17)
90 days					
All individuals	0.35 (0.30-0,39)	0.34 (0.29-0.38)	0.01 (-0.05-0.07)	9930 (1483-∞)	1.03 (0.87-1.22)
Women	0.30 (0.25-0.35)	0.31 (0.25-0.36)	0.00 (-0.08-0.07)	-20325 (1417- -1243)	0.98 (0.77-1.27)

© 2026 Bergh Fahlén C et al. *JAMA Network Open.*

Men	0.36 (0.30-0.42)	0.36 (0.30-0.41)	0.01 (-0.08-0.09)	20086 (1118-∞)	1.01 (0.80-1.29)
<65	0.11 (0.08-0.14)	0.13 (0.10-0.17)	-0.02 (-0.07-0.02)	-4270 (4351- -1432)	0.82 (0.55-1.24)
65–79	0.30 (0.24-0.37)	0.35 (0.28-0.42)	-0.05 (-0.14-0.05)	-2116 (2097- -703)	0.86 (0.64-1.17)
≥80	1.56 (1.30-1.83)	1.33 (1.09-1.58)	0.23 (-0.13-0.59)	433 (171-∞)	1.17 (0.91-1.50)
Women <65	0.08 (0.04-0.11)	0.07 (0.03-0.11)	0.01(-0.05-0.06)	19979 (1727-∞)	1.07 (0.46-2.44)
Women 65-79	0.20 (0.13-0.27)	0.29 (0.20-0.37)	-0.09 (-0.20-0.02)	-1130 (4281- - 499)	0.69 (0.43-1.16)
Women ≥80	1.56 (1.24-1.88)	1.21 (0.92-1.48)	0.35 (-0.08-0.78)	284 (129-∞)	1.29 (0.93-1.77)
Men <65	0.12 (0.08-0.17)	0.14 (0.09-0.19)	-0.02 (-0.09-0.05)	-5684 (2017- -1180)	0.88 (0.52-1.52)
Men 65-79	0.42 (0.31-0.53)	0.43 (0.32-0.54)	-0.01 (-0.17-0.15)	-11854 (663- -596)	0.98 (0.67-1.45)
Men ≥80	1.81 (1.32-2.29)	1.67 (1.20-2.14)	0.14 (-0.54-0.81)	743 (124-∞)	1.08 (0.72-1.61)
730 days					
All individuals	2.73 (2.62-2.85)	2.98 (2.86-3.10)	-0.24 (-0.41- -0.08)	-410 (-1311- -243)	0.92 (0.87-0.97)
Women	2.59 (2.43-2.75)	2.96 (2.79-3.13)	-0.37 (-0.60- -0.14)	-268 (-696- -166)	0.87 (0.80-0.95)
Men	2.68 (2.51-2.84)	2.96 (2.79-3.14)	-0.29 (-0.53- -0.05)	-351 (-2193- -191)	0.90 (-0.83-0.98)
<65	0.87 (0.78-0.96)	1.13 (1.03-1.24)	-0.26 (-0.4- -0.13)	-380 (-791- -250)	0.77 (0.67-0.88)
65–79	2.92 (2.71-3.12)	2.23 (3.02-3.45)	-0.32 (-0.61- -0.02)	-315 (-4350- -164)	0.90 (0.82-0.99)
≥80	10.89 (10.22-11.56)	11.24 (10.56 -11.91)	-0.35 (-1.3-0.61)	-290 (166- -78)	0.97 (0.89-1.06)
Women <65	0.64 (0.52-0.75)	0.85 (0.72-0.98)	-0.22 (-0.39- -0.04)	-463 (-2296- -257)	0.75 (0.59-0.95)
Women 65-79	2.29 (2.05-2.54)	2.88 (2.60-3.15)	-0.58 (-0.95- -0.20)	-172 (-467- -105)	0.80 (0.69-0.92)
Women ≥80	10.84 (10.02-11.65)	10.06 (9.26-10.84)	0.78 (-0.35-1.92)	128 (52-∞)	1.08 (0.97-1.20)
Men <65	1.13 (0.99-1.28)	1.31 (1.16-1.46)	-0.18 (-0.39- -0.028)	-559 (3529- -259)	0.86 (0.73-1-03)
Men 65-79	3.38 (3.06-3.69)	3.61 (3.28-3.95)	-0.24 (-0.70-0.22)	-417 (456- -143)	0.93 (0.82-1.07)
Men ≥80	12.09 (10.87-13.30)	13.82 (12.52-15.10)	-1.73 (-3.50-0.041)	-58 (2468- -29)	0.88 (0.76-1.00)

[1]CI
[2]Calcium Channel Blockers
[3]Relativ risks

© 2026 Bergh Fahlén C et al. *JAMA Network Open.*

eFigure. Study Flow Diagram of the Study Population

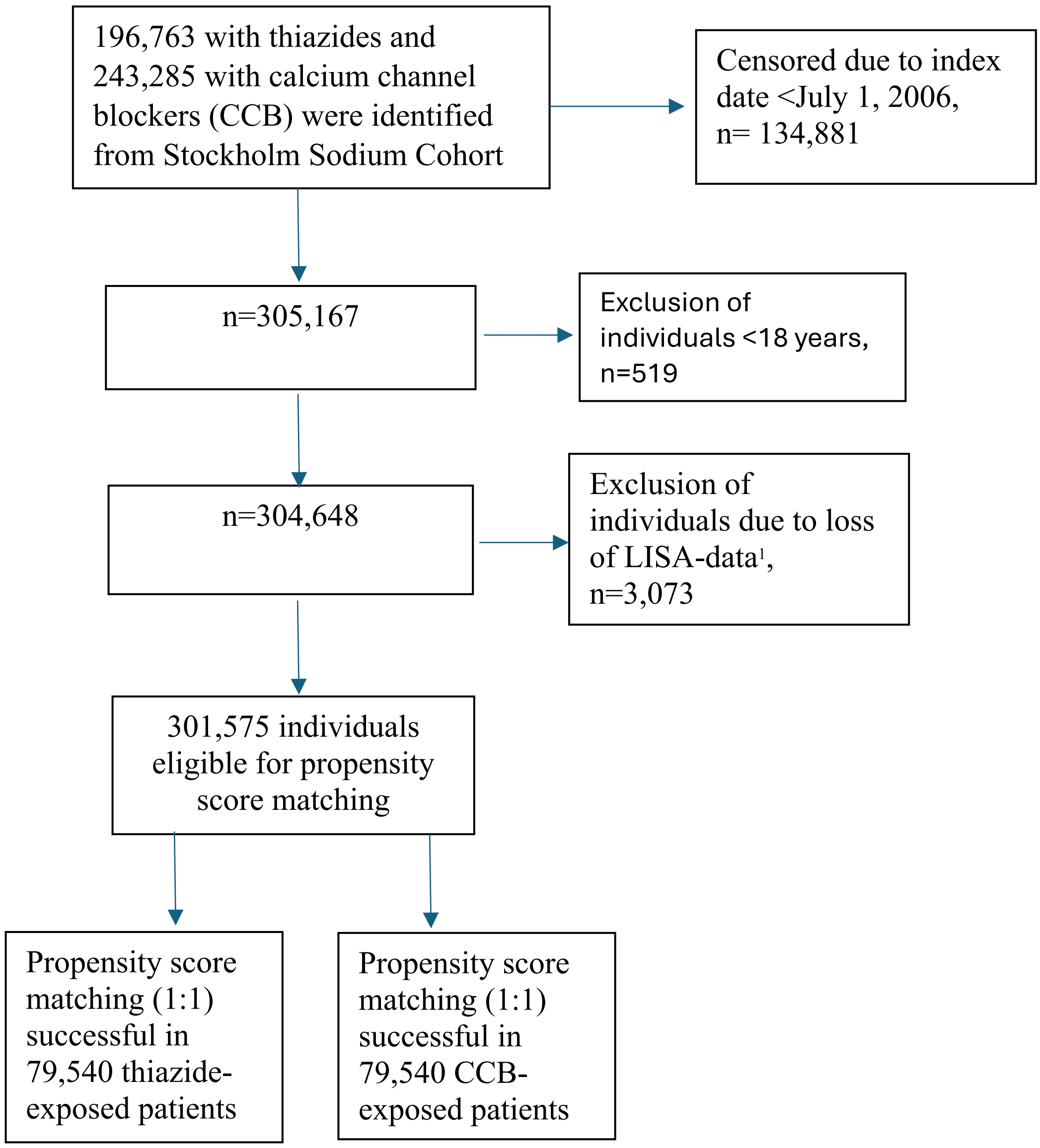

[1]Data on education, employment and income collected from the longitudinal integrated database for health insurance and labor market studies

© 2026 Bergh Fahlén C et al. *JAMA Network Open.*
