# Supplementary material for: Thiazides and Risk of Hyponatremia by Age and Sex
Source: JAMA Netw Open. 2026 Apr 2;9(4):e264642. doi: 10.1001/jamanetworkopen.2026.4642 (PMC13047463; doi:10.1001/jamanetworkopen.2026.4642)
Supplement: Supplement 2. — Data Sharing Statement [file jamanetwopen-e264642-s002.pdf]

Data Sharing Statement

Bergh Fahlén. Thiazides and Risk of Hyponatremia by Age and Sex. *JAMA Netw Open*. Published April 02, 2026. doi:10.1001/jamanetworkopen.2026.4642

Data
Data available: No
